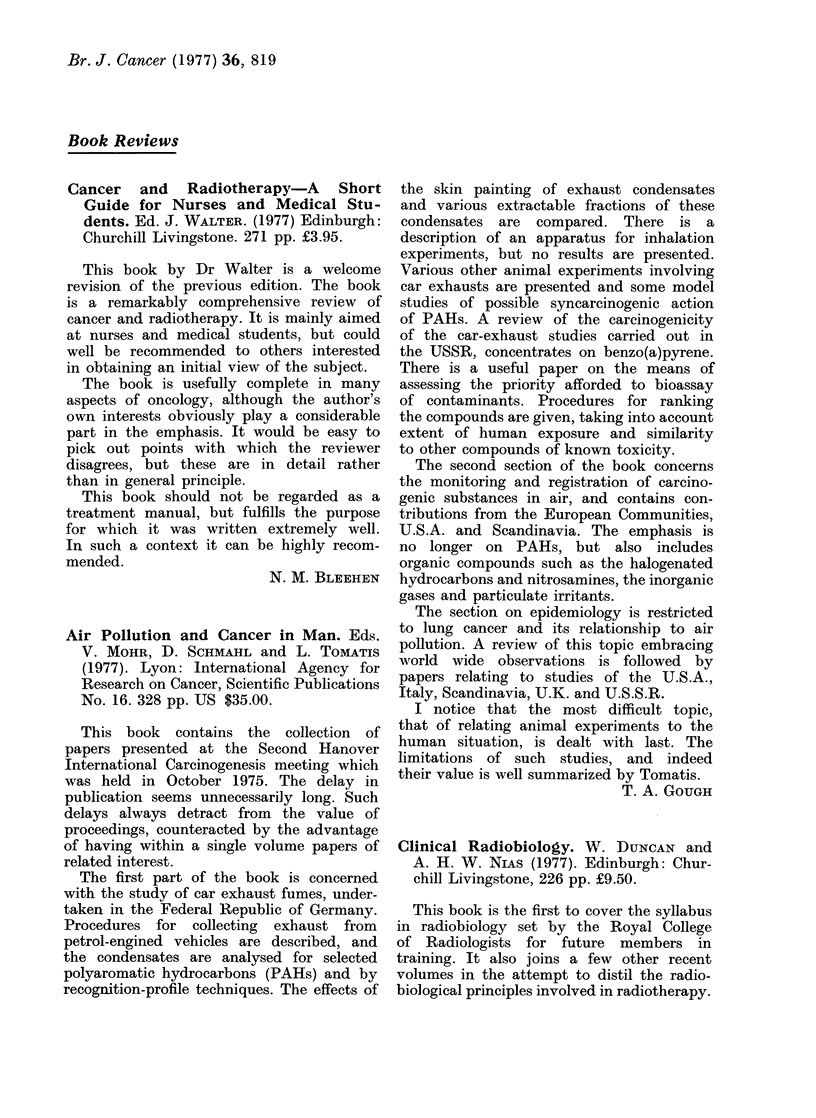# Air Pollution and Cancer in Man

**Published:** 1977-12

**Authors:** T. A. Gough


**Air Pollution and Cancer in Man.** Eds. V. MOHR, D. SCHMAHL and L. TOMATIS (1977). Lyon: International Agency for Research on Cancer, Scientific Publications No. 16. 328 pp. US $35.00.

This book contains the collection of papers presented at the Second Hanover International Carcinogenesis meeting which was held in October 1975. The delay in publication seems unnecessarily long. Such delays always detract from the value of proceedings, counteracted by the advantage of having within a single volume papers of related interest.

The first part of the book is concerned with the study of car exhaust fumes, undertaken in the Federal Republic of Germany. Procedures for collecting exhaust from petrol-engined vehicles are described, and the condensates are analysed for selected polyaromatic hydrocarbons (PAHs) and by recognition-profile techniques. The effects of the skin painting of exhaust condensates and various extractable fractions of these condensates are compared. There is a description of an apparatus for inhalation experiments, but no results are presented. Various other animal experiments involving car exhausts are presented and some model studies of possible syncarcinogenic action of PAHs. A review of the carcinogenicity of the car-exhaust studies carried out in the USSR, concentrates on benzo(a)pyrene. There is a useful paper on the means of assessing the priority afforded to bioassay of contaminants. Procedures for ranking the compounds are given, taking into account extent of human exposure and similarity to other compounds of known toxicity.

The second section of the book concerns the monitoring and registration of carcinogenic substances in air, and contains contributions from the European Communities, U.S.A. and Scandinavia. The emphasis is no longer on PAHs, but also includes organic compounds such as the halogenated hydrocarbons and nitrosamines, the inorganic gases and particulate irritants.

The section on epidemiology is restricted to lung cancer and its relationship to air pollution. A review of this topic embracing world wide observations is followed by papers relating to studies of the U.S.A., Italy, Scandinavia, U.K. and U.S.S.R.

I notice that the most difficult topic, that of relating animal experiments to the human situation, is dealt with last. The limitations of such studies, and indeed their value is well summarized by Tomatis.

T. A. GOUGH